# The Therapeutic Effectiveness of Neoadjuvant Trastuzumab Plus Chemotherapy for HER2-Positive Breast Cancer Can Be Predicted by Tumor-Infiltrating Lymphocytes and PD-L1 Expression

**DOI:** 10.3389/fonc.2021.706606

**Published:** 2022-01-05

**Authors:** Mao Shang, Yajing Chi, Jianbo Zhang, Jin Chang, Hui Yang, Sha Yin, Qiaorui Tan, Xiaochu Man, Huihui Li

**Affiliations:** ^1^ Department of Breast Medical Oncology, Shandong Cancer Hospital and Institute, Shandong First Medical University & Shandong Academy of Medical Sciences, Jinan, China; ^2^ Department of Oncology, Jinan Central Hospital, Shandong First Medical University & Shandong Academy of Medical Sciences, Jinan, China; ^3^ Department of Pathology, Shandong Cancer Hospital and Institute, Shandong First Medical University & Shandong Academy of Medical Sciences, Jinan, China; ^4^ Department of Radiation Oncology, The Second Affiliated Hospital of Shandong First Medical University, Taian, China; ^5^ Department of Pathology, The Second Affiliated Hospital of Shandong First Medical University, Taian, China

**Keywords:** HER2-positive breast cancer, neoadjuvant trastuzumab plus chemotherapy, therapeutic effect, PD-L1, TILs

## Abstract

**Introduction:**

Neoadjuvant trastuzumab plus chemotherapy may affect programmed death-ligand 1 (PD-L1) expression and tumor-infiltrating lymphocytes (TILs) in HER2-positive breast cancer. Discordant results were shown on the correlation between PD-L1 expression or TILs and the effectiveness of neoadjuvant therapy in HER2-positive breast cancer patients. This study aimed to clarify the predictive value of PD-L1 expression and TILs in neoadjuvant therapy in patients with HER2-positive breast cancer.

**Methods:**

HER2-positive breast cancer cases receiving neoadjuvant treatment (NAT; *n* = 155) were retrospectively collected from July 2013 to November 2018. Histopathologic analysis of TILs was performed on hematoxylin and eosin (H&E)-stained sections from pre- and post-NAT specimens. The TIL score as a categorical variable can be divided into high (≥30%) and low (<30%) categories. The expression of PD-L1 was detected by immunohistochemistry, and the percentage of positive membranous staining (at least 1%) in tumor cells (PD-L1+TC) and TILs (PD-L1+TILs) was scored.

**Results:**

In our study, 87 patients received neoadjuvant chemotherapy alone and 68 received neoadjuvant trastuzumab plus chemotherapy. Multivariate logistic regression analysis confirmed that lymph node metastasis, high TILs, and PD-L1+TILs in pre-neoadjuvant therapy specimens were independent predictors of pathological complete response (pCR) in neoadjuvant therapy (*p* < 0.05, for all). Among all patients, TILs were increased in breast cancer tissues post-neoadjuvant therapy (*p* < 0.001). Consistent results were found in the subgroup analysis of the trastuzumab plus chemotherapy group and the chemotherapy alone group (*p* < 0.05, for both). In 116 non-pCR patients, PD-L1+TC was decreased in breast cancer tissues post-neoadjuvant therapy (*p* = 0.0219). Consistent results were found in 43 non-pCR patients who received neoadjuvant trastuzumab plus chemotherapy (*p* = 0.0437). However, in 73 non-pCR patients who received neoadjuvant chemotherapy, there was no significant difference in PD-L1+TC expression in pre- and post-neoadjuvant therapy specimens (*p* = 0.1465). On the other hand, in the general population, the neoadjuvant trastuzumab plus chemotherapy group, and the neoadjuvant chemotherapy group, PD-L1+TILs decreased after treatment (*p* < 0.05, for both).

**Conclusion:**

Higher TIL counts and PD-L1+TILs in pre-neoadjuvant therapy specimens and lymph node metastasis are independent predictors of pCR in patients with HER2-positive breast cancer treated with neoadjuvant therapy. TIL counts, PD-L1+TC, and PD-L1+TILs changed before and after neoadjuvant trastuzumab plus chemotherapy for HER2-positive breast cancer, which may suggest that, in HER2-positive breast cancer, neoadjuvant trastuzumab plus chemotherapy may stimulate the antitumor immune effect of the host, thereby preventing tumor immune escape.

## Introduction

Trastuzumab plus pertuzumab and chemotherapy have been confirmed as the neoadjuvant therapy for stage II–III HER2-positive breast cancer (1). Before 2020, since pertuzumab was not included in medical insurance, many patients with HER2-positive breast cancer still choose trastuzumab plus chemotherapy as neoadjuvant treatment. In HER2-positive breast cancer, neoadjuvant trastuzumab plus chemotherapy can dramatically increase effectiveness compared to chemotherapy alone. However, there were still 25% of patients who showed tumor progression after treatment, thus affecting the prognosis (1–4). Therefore, there is an urgent need to find an accurate and reliable biomarker to predict who will benefit from this treatment. Up to now, several clinical factors, such as lymph node metastasis, tumor size, and hormone receptor (HR) expression, have been correlated with the efficacy of neoadjuvant treatment (NAT) (5, 6). However, choosing NAT based on the above factors does not benefit all patients. Therefore, a molecular marker that can reliably and efficiently assess the effectiveness of NAT is critical in HER2-positive breast cancer.

Programmed death-ligand 1 (PD-L1) is a B7 immune molecule transmembrane protein found in several tumor cells and immune cells, which mediates tumor immunosuppression and is linked to tumor cell immune escape (7, 8). Research shows that trastuzumab can affect PD-L1 expression on CD8^+^ T cells and cancer cells in HER2-positive breast cancer (9–11). However, another study observed that trastuzumab could downregulate the effects of PD-L1 on cancer cells through HER2 inhibition (10). Furthermore, the PANACEA trial also proposed the hypothesis that trastuzumab can reverse tumor-mediated immunosuppression and activate the local antitumor immune effect (12). Chemotherapy can also cause immunogenic cell death and cellular damage (13). However, the impact of PD-L1 expression on cancer cells and lymphocytes in HER2-positive breast cancer remains unknown.

Tumor-infiltrating lymphocytes (TILs), as immune cells that penetrate tumor tissues, may be associated with immune-mediated tumor–host interaction and antibody-dependent cell-mediated cytotoxicity (ADCC) (14–16). Previous research suggested that increased baseline TILs in patients may be related to the benefit of the anti-HER2 monoclonal antibody trastuzumab (17–20). However, another study showed that high levels of TILs were linked to a lack of benefits from trastuzumab therapy (21). Consequently, the effects of TILs on neoadjuvant trastuzumab plus chemotherapy on HER2-positive breast cancer patients remain a mystery.

Recent research has shown that the active HER2 oncogene regulates the mobilization and activation of tumor-infiltrating immune cells and the therapeutic activity of trastuzumab (22, 23). In several trials, elevated levels of TILs were linked to the benefits of trastuzumab plus chemotherapy. However, experiments on the predictive value of PD-L1 and TILs in the effectiveness of NAT for HER2-positive breast cancer patients showed discordant results. They mainly emphasized the correlation between the expression of PD-L1 or TILs and the efficiency of NAT in the tissue; however, there were no differences in the harmonizing tissues prior to and following NAT.

This research aimed to investigate how TILs and PD-L1 expression in paired tissues changed prior to and following NAT, as well as the relationship between these improvements and the effectiveness of neoadjuvant trastuzumab plus chemotherapy in HER2-positive breast cancer patients.

## Materials and Methods

### Patients

**Figure 1:**
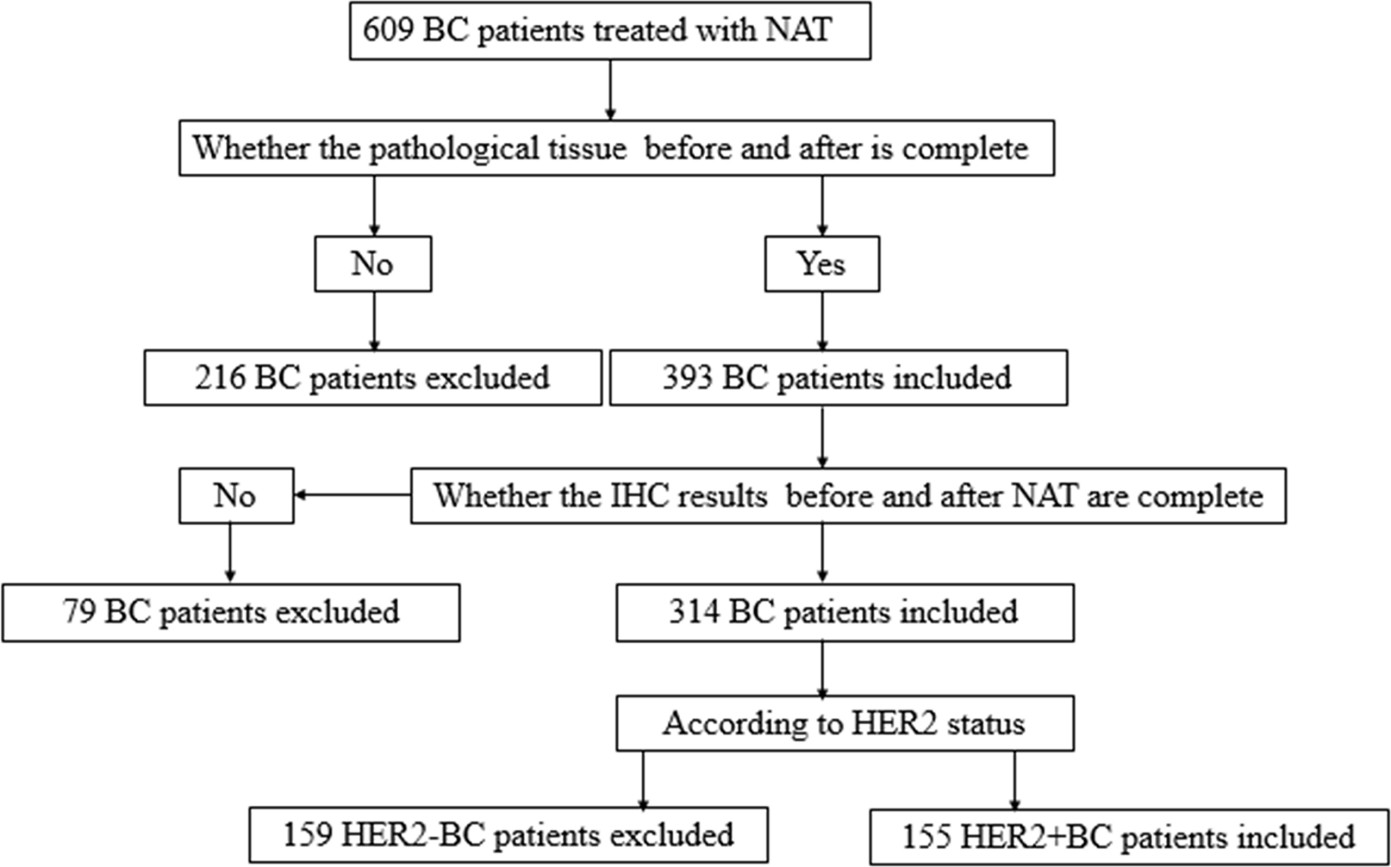
Flowchart explaining the process of patient selection.

Data were obtained from 155 cases of HER2-positive invasive breast cancer patients at the Shandong Cancer Hospital from July 2013 to November 2018. Diagnosis of patients was confirmed histologically by core needle biopsy, and the stage of the disease was clarified using ultrasonography (US), bone scintigraphy, and computed tomography (CT). The medical and pathology records of these patients were examined through the hospital medical record system. A flowchart summarizing the patient selection process followed is shown in **Figure 1**. We accessed formalin-fixed, paraffin-embedded tissue samples from NAT patients. A proportion of patients receiving NAT were treated with a taxane-containing regimen along with platinum or an anthracycline. Another proportion of patients received anti-HER2 trastuzumab combined with chemotherapy. The following clinicopathologic variables were acquired: age, tumor dimension, status of the lymph node, initial HR, Ki-67 proliferation index, and neoadjuvant therapy (with and without trastuzumab). Pathological complete response (pCR) was identified as noninvasive breast cancer and axillary lymph nodes remaining after NAT (ypT0 ypN0).

### Immunohistochemistry

Immunohistochemical staining was performed on 155 paired breast cancer surgery and biopsy samples with 3.7% neutral formaldehyde, the samples were embedded in paraffin, and 4-μm-thick serial parts were fixed to the samples. This was followed by xylene dewaxing, ethanol graded hydration, ethylenediaminetetraacetic acid (EDTA) antigen repair solution, phosphate-buffered saline (PBS) rinsing (1:50 dilution; clone SP142, Ventana, Shanghai Roche Diagnostic Products Limited Company, Shanghai, China) overnight in a 37°C incubator, treatment with goat anti-mouse/rabbit IgG polymer secondary antibody dropwise, and 3,3′-diaminobenzidine (DAB) development. Contrast dyeing of hematoxylin was performed, followed by ethanol dehydration and sealing. Immunophenotyping was carried out using the following antibodies: anti-ER (clone 6F11; Leica Microsystems, Bannockburn, IL, USA), anti-PR (clone 16; Leica Microsystems), anti-HER2/neu (Ventana 4B5; Ventana Medical Systems, Tucson, AZ, USA), and Ki-67 (MIB-1; Ventana Medical Systems). Estrogen receptor (ER) and progesterone receptor (PR) positivity was defined as staining of ≥1% tumor cell nuclei, while HER2 positivity was evaluated following the American Society of Clinical Oncology (ASCO)/College of American Pathologists (CAP) criteria (24). Briefly, sections with a HER2/CEP17 ratio of ≥2.0 and copy number ≥4.0 or a dual-probe HER2/CEP17 ratio of <2.0 with ≥6.0 HER2 signal per nucleus were determined as positive. A HER2-negative status was defined as a HER2/CEP17 ratio ≥2.0 with <4.0 HER2 signal per nucleus, or a HER2/CEP17 ratio <2.0 and ≥4.0 + <6.0 HER2 signal per nucleus, or a HER2/CEP17 ratio <2 and <4.0 HER2 signal per nucleus. The Ki-67 status was determined by analyzing at least 500 cancer cells per patient. Five high-power-field images were used for each section. Patients were categorized into three groups based on the percentages of Ki-67-positive tumor cells: low, <15%; intermediate, 15%–30%; and high, >30% (25).

### PD-L1 Immunohistochemistry

**Figure 2:**
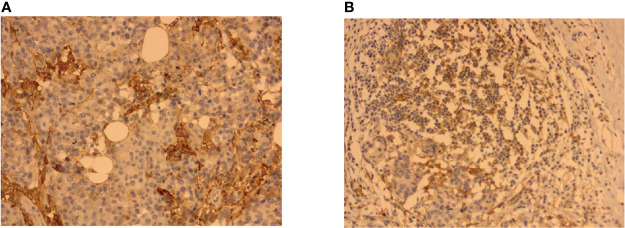
Representative immunohistochemistry (IHC) images showing PD-L1+TC and PD-L1+TILs in breast cancer tissues. **(A)** PD-L1 immunostaining on tumor cells (TC). **(B)** PD-L1 immunostaining on tumor-infiltrating lymphocytes (TILs).

In this study, a two-step immunohistochemistry method was used. The PD-L1 antibody reagent is a rabbit monoclonal antibody (ZZR3). PD-L1 on the tumor cell (TC) membrane or cytoplasm was recorded as PD-L1+TC, and the expression on TILs was recorded as PD-L1+TILs (**Figure 2**). The monoclonal antibody was used to stain breast cancer pathological sections using established methods (26, 27). A 5% increase from 0% to 100% was observed in carcinoma cells with direct membrane PD-L1 expression; less than 1% had a negative markup. For each tumor, the mean PD-L1 labeling was calculated across all cells (28). PD-L1 expression (in percentage) by TILs was also documented in 5% increments and scored as none (0), focal (1+; <5%), moderate (2+; 5%–50%), or severe (3+; 51%–100%). If the PD-L1-positive membrane staining percentage scores of TC and TILs in the tissue after treatment were lower than those before NAT, it was defined as a decrease in PD-L1+TC or a decrease in PD-L1+TILs; otherwise, it was defined as an increase in PD-L1+TC or PD-L1+TIL.

### Assessment of TILs

TILs were histopathologically examined using H&E-stained portions from 155 breast cancer samples. The tumor bed was tested and graded in those cases that achieved pCR. Two pathologists (YSG and HY) blinded to the clinical criteria and reactions evaluated the TILs separately. All mononuclear cells including lymphocytes and plasma cells were graded, except granulocytes and other polymorphonuclear leukocytes; invasive lesions and inflammatory infiltration in the matrix of normal breast structures were excluded (29). The TIL count was defined as a percentage estimate of the stromal area adjacent to the tumor that contained mononuclear cells (30). When the TIL score was used as a categorical variable, it was divided into two categories: high TILs (≥30%) and low TILs (<30%) (29). The TIL count of post-NAT surgical resection tissue minus the TIL count of pre-neoadjuvant therapy core needle biopsies represents the change in TILs. If the TIL count of the tissue after treatment was increased compared with that before treatment, it was defined as an increase in TILs; otherwise, it was defined as a decrease in TILs.

### Statistical Analysis

The chi-square test was employed to assess the relationships between PD-L1+TC, PD-L1+TILs, TILs, and patients’ clinicopathological characteristics. To determine the variables that were substantially correlated with pCR, we applied univariate and multivariate logistic regression analyses. Wilcoxon’s non-parametric test was used to compare the changes between the values of PD-L1+TC, PD-L1+TILs, and TILs before and after neoadjuvant treatment. The relationship between PD-L1+TC, PD-L1+TILs, TILs, and disease-free survival (DFS) was determined using the Kaplan–Meier procedure, and the results were compared using the log-rank test. The Cox regression model was adopted to conduct a multivariate study of the prognostic variables. SPSS version 22 was used for all analyses (IBM Corp., Armonk, NY, USA). A *p* < 0.05 was considered statistically significant.

## Results

### Patients’ Characteristics

One hundred and fifty-five HER2-positive breast cancer patients were included in this study. The characteristic features of the study population are listed in **Table 1**. The median age of the patients was 50 years (range = 28–74 years). Most of the patients were older than 50 years (55.5%) at the time of diagnosis. Of the patients, 71 (45.8%) were menopausal. There were 126 (81.3%) patients with a clinical tumor diameter larger than 2.0 cm and 148 (93.5%) patients with clinical lymph node metastases. Sixty-eight patients (43.9%) received neoadjuvant trastuzumab plus chemotherapy, while 87 (56.1%) received neoadjuvant chemotherapy. According to the Miller–Payne scoring system, 39 (25.2%) patients realized pCR and 116 (74.8%) patients were non-pCR.

**Table 1:** Patient characteristics.

Characteristics	*N* (%)
Age (years)	
≤50	69 (44.5)
>50	86 (55.5)
Menstrual status	
Menopause	71 (45.8)
Non-menopausal	84 (54.2)
cT (cm)	
≤2	29 (18.7)
>2	126 (81.3)
cN	
Negative	7 (4.5)
Positive	148 (93.5)
Neoadjuvant treatment	
Trastuzumab+chemotherapy	68 (43.9)
Chemotherapy	87 (56.1)
Neoadjuvant efficacy	
pCR	39 (25.2)
No pCR	116 (74.8)

cT, primary tumor; cN, lymph node involvement; pCR, pathological complete response.

### Expressions of TILs, PD-L1+TC, and PD-L1+TILs in Samples Before and After Neoadjuvant Treatment and their Correlations With Clinicopathological Characteristics

As shown in **Tables 2** and **3**, in samples before neoadjuvant therapy, TILs were negatively associated with the expression of PR, while PD-L1+TILs were negatively associated with the expressions of ER and PR (*p* < 0.05, for all). However, in samples before neoadjuvant therapy, no correlation between PD-L1+TC and age, primary tumor (cT), lymph node involvement (cN), ER status, PR status, or Ki-67 index was found (*p* > 0.05). In samples after neoadjuvant therapy, the expression of PD-L1+TC was negatively correlated with ER (*p* < 0.05). As for the TILs and PD-L1+TILs in samples after neoadjuvant therapy, there was no correlation with age, cT, cN, ER status, PR status, or Ki-67 index.

**Table 2:** Expressions of TILs, PD-L1+TC, and PD-L1+TILs in pre-neoadjuvant therapy specimens and correlations with clinicopathological characteristics.

	TILs			PD-L1+TC Status			PD-L1+TILs		
	High	Low	*p*-value	Positive	Negative	*p*-value	Positive	Negative	*p*-value
Age (years)			0.711			0.598			0.348
≤50	6	63		43	26		22	45	
>50	9	77		50	36		22	63	
cN			0.999			0.155			0.660
Negative	0	7		6	1		1	1	
Positive	15	133		87	61		61	43	
cT (cm)			0.208			0.501			0.961
≤2	1	47		19	10		8	20	
>2	14	62		74	52		36	88	
ER			0.446			0.389			0.001
Negative	5	61		37	29		9	55	
Positive	10	79		56	33		35	53	
PR			0.004			0.749			0.009
Negative	5	85		55	35		18	69	
Positive	10	55		38	27		26	39	
Ki-67 index			0.110			0.249			0.469
Low	0	9		6	3		4	5	
Intermediate	3	61		43	21		19	44	
High	2	68		43	37		20	58	

TILs, tumor-infiltrating lymphocytes; PD-L1, programmed death-ligand 1; cN, lymph node involvement; cT, primary tumor; ER, estrogen receptor; PR, progesterone receptor.

**Table 3:** Expressions of TILs, PD-L1+TC, and PD-L1+TILs in post-neoadjuvant therapy specimens and correlations with clinicopathological characteristics.

	TILs			PD-L1+TC Status			PD-L1+TILs		
	High	Low	*p*-value	Positive	Negative	*p*-value	Positive	Negative	*p*-value
Age (years)			0.526			0.892			0.193
≤50	13	47		39	12		37	22	
>50	13	62		49	16		37	35	
cN			0.620			0.426			0.971
Negative	2	5		1	1		4	3	
Positive	24	104		87	27		70	54	
cT (cm)			0.997			0.556			0.102
≤2	5	21		15	3		19	8	
>2	21	88		73	25		55	49	
ER			0.583			0.012			0.059
Negative	13	48		30	17		28	31	
Positive	13	61		58	11		46	26	
PR			0.128			0.351			0.784
Negative	13	72		51	19		45	36	
Positive	13	37		37	9		29	21	
Ki-67 index			0.889			0.211			0.413
Low	2	7		9	0		7	2	
Intermediate	10	46		36	14		28	23	
High	14	54		41	14		38	31	

TILs, tumor-infiltrating lymphocytes; PD-L1, programmed death-ligand 1; cN, lymph node involvement; cT, primary tumor; ER, estrogen receptor; PR, progesterone receptor.

### Correlation of the Expressions of TILs, PD-L1+TC, and PD-L1+TILs With Clinicopathological Factors Including pCR to Neoadjuvant Therapy

As shown in **Table 4**, univariate analysis confirmed that pCR had a positive correlation with cN, high TIL counts, and PD-L1+TILs in specimens prior to neoadjuvant therapy (*p* < 0.05, for all), but not with age, menstruation, cN, cT, ER, PR, and Ki-67 index in PD-L1+TC before neoadjuvant therapy, PD-L1+TILs after neoadjuvant therapy, TIL changes, or PD-L1+TIL changes (*p* > 0.05, for all). Multivariate logistic regression analysis verified that cN, high TIL counts, and PD-L1+TILs in pre-NAT samples were significantly correlated with pCR (*p* < 0.05, for all).

**Table 4:** Correlation of the expressions of TILs, PD-L1+TC, and PD-L1+TILs with clinicopathological factors including pCR to neoadjuvant therapy.

Parameters	Univariate			Multivariate		
	Hazard Ratio	95%CI	*p*-value	Hazard Ratio	95%CI	*p*-value
Age (years)	1.048	0.489–2.244	0.905			
≤50						
>50						
Menstruation	1.349	0.626–2.911	0.445			
Menopausal						
Non-menopausal						
cN	7.344	1.358–39.727	0.021	0.115	0.020–0.659	0.015
Negative						
Positive						
cT (cm)	1.966	0.817–4.732	0.131			
≤2						
>2						
ER	0.326	0.619–2.841	0.467			
Negative						
Positive						
PR	0.553	0.256–1.194	0.132			
Negative						
Positive						
Ki-67 index	0.609	0.277–1.338	0.466			
Low Intermediate High						
Pretreatment PD-L1+TC	0.635	0.289–1.396	0.258			
Negative						
Positive						
Pretreatment TILs	0.091	0.018–0.463	0.004	0.102	0.020–0.533	0.007
Low						
High						
Pretreatment PD-L1+TILs	0.202	0.066–0.620	0.005	0.272	0.085–0.872	0.028
Negative						
Positive						
TIL change	1.875	0.668–5.266	0.233			
Decreased						
Increased						
Unchanged						
PD-L1+TIL change	3.967	1.051–14.970	0.089			
Decreased						
Increased						
Unchanged						

TILs, tumor-infiltrating lymphocytes; PD-L1, programmed death-ligand 1; cN, lymph node involvement; cT, primary tumor; ER, estrogen receptor; PR, progesterone receptor.

### Changes in TILs, PD-L1+TC, and PD-L1+TILs Before and After Neoadjuvant Treatment

**Figure 3:**
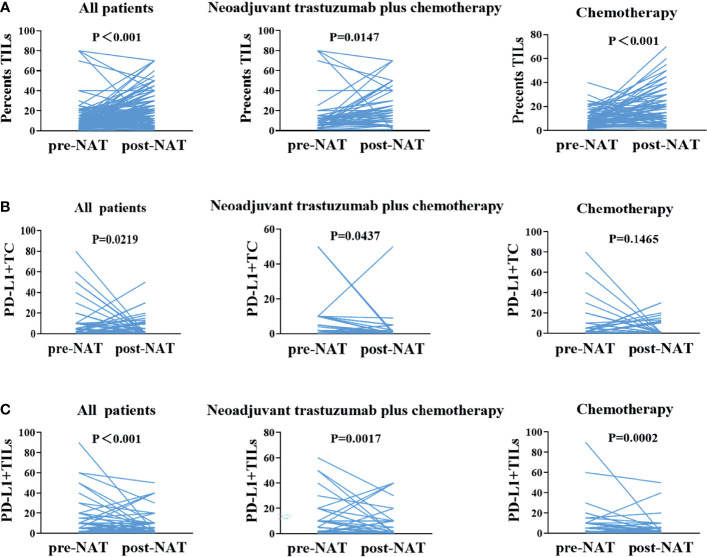
One-to-one correspondence of tumor-infiltrating lymphocytes (TILs), PD-L1+TC, and PD-L1+TILs between pre- and post-neoadjuvant therapy samples for all cases. **(A)** Changes in TILs between before and after neoadjuvant therapy, before and after neoadjuvant trastuzumab plus chemotherapy, and before and after neoadjuvant chemotherapy. **(B)** In non-pCR patients, changes in PD-L1+TC between pre- and post-neoadjuvant therapy, before and after neoadjuvant trastuzumab plus chemotherapy, and before and after neoadjuvant chemotherapy. **(C)** Changes in PD-L1+TILs between pre- and post-neoadjuvant therapy and before and after neoadjuvant trastuzumab plus chemotherapy. *pCR*, pathological complete response.

The TIL counts in breast cancer tissues improved after neoadjuvant therapy in all patients (*p* < 0.001). Subgroup analysis of the trastuzumab plus chemotherapy group and the chemotherapy alone group revealed consistent findings (*p* < 0.05). The expressions of PD-L1+TC was reduced in breast cancer tissues after NAT in 116 non-pCR patients (*p* = 0.0219). In 43 non-pCR patients who received neoadjuvant trastuzumab plus chemotherapy, consistent findings were observed (*p* = 0.0437). Although neoadjuvant chemotherapy was given to 73 non-pCR patients, there was no substantial difference in PD-L1+TC expression before and after neoadjuvant therapy (*p* = 0.1465). PD-L1+TILs were downregulated following treatment in the general population, the neoadjuvant trastuzumab plus chemotherapy group, and the neoadjuvant chemotherapy group (*p* < 0.05; **Figure 3**).

### Relationship Between Changes in Various Factors and Prognosis

**Figure 4:**
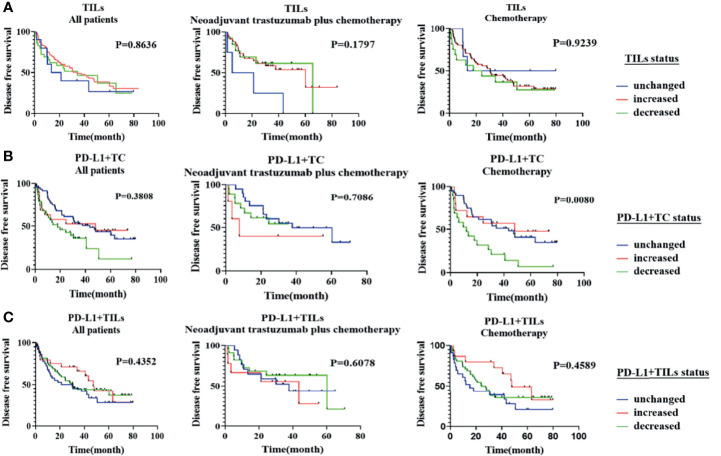
Kaplan–Meier analysis of disease-free survival (DFS) according to the changes in tumor-infiltrating lymphocytes (TILs), PD-L1+TC, and PD-L1+TILs between pre- and post-neoadjuvant treatment. **(A)** The changes in TILs before and after neoadjuvant treatment were not significantly correlated with patients’ DFS in all populations, the neoadjuvant trastuzumab plus chemotherapy group, and the neoadjuvant chemotherapy group (*p* > 0.05, for all). **(B)** The changes in PD-L1+TC before and after neoadjuvant chemotherapy were related to DFS (*p* = 0.0080), but the changes in PD-L1+TC in all populations and the neoadjuvant trastuzumab plus chemotherapy population were not related to DFS (*p* > 0.05, for all). **(C)** The changes in PD-L1+TILs before and after neoadjuvant treatment were not significantly correlated with patients’ DFS in all populations, the neoadjuvant trastuzumab plus chemotherapy group, and the neoadjuvant chemotherapy group (*p* > 0.05, for all).

According to the Kaplan–Meier study, only the changes in PD-L1+TC before and after neoadjuvant chemotherapy were related to DFS (*p* = 0.0080). Nevertheless, the transition in TILs and PD-L1+TILs between pre and post-NAT showed no association with DFS (*p* > 0.05; **Figure 4**). A multivariate Cox regression study was performed using the significant clinicopathological variables identified by univariate analysis (cN, cT, and PD-L1+TC before treatment). We did not find the above factors to be independent predictors of DFS (*p* > 0.05, for all; **Table 5**).

**Table 5:** Factors correlated with disease-free survival (DFS) in univariate and multivariate analyses.

Clinicopathological Parameters	DFS					
	Univariate Analysis			Multivariate Analysis		
	HR	95%CI	*p*-value	HR	95%CI	*p*-value
cN	0.363	0.089–1.477	0.157	0.491	0.117–2.060	0.331
cT	0.543	0.295–1.002	0.051	0.594	0.318–1.108	0.102
ER	1.156	0.757–1.763	0.502			
PR	1.077	0.699–1.659	0.736			
Ki-67 index	1.037	0.467–2.302	0.378			
Pretreatment PD-L1+TILs	1.009	0.635–1.604	0.969			
Pretreatment TILs	1.320	0.531–3.283	0.550			
Pretreatment PD-L1+TC	0.720	0.463–1.120	0.145	0.744	0.478–1.159	0.191
TIL change	0.623	0.209–1.860	0.808			
PD-L1-TIL change	0.769	0.468–1.266	0.380			

TILs, tumor-infiltrating lymphocytes; PD-L1, programmed death-ligand 1; cN, lymph node involvement; cT, primary tumor; ER, estrogen receptor; PR, progesterone receptor.

## Discussion

Numerous experiments have been conducted to investigate the predictors of NAT effectiveness in HER2-positive breast cancer. Until now, no accurate and commonly used biomarker has been discovered, except for a few clinicopathological factors such as HER2. The HER2 oncogene can affect the therapeutic effect of trastuzumab by inducing the expression of PD-L1 and the recruitment and activation of TILs, suggesting that TILs and PD-L1 have been linked to trastuzumab efficacy (9, 21, 23, 31). Several studies have confirmed that TILs and PD-L1 have such predictive values in HER2-positive breast cancer patients, but debate is still ongoing (32–34). Most of the previous studies have concentrated on the expression of PD-L1 or TILs in tissues before NAT in HER2-positive breast cancer to predict the effectiveness of neoadjuvant therapy. There is still lack of information on whether the changes in PD-L1 and TILs in the tissues before and after NAT could predict the efficacy of neoadjuvant treatment.

We first tested whether there was any association between the TIL counts and clinicopathological characteristics before and after neoadjuvant therapy. Previous studies have shown that higher TIL counts pre-NAT were significantly associated with more aggressive clinicopathological features, such as higher cT staging, histological grade, and Ki-67 index (35). In our study, we concluded that the TIL counts in tissues before NAT were significantly higher in PR-negative cases. Consistent with previous research (36), no evidence of an association was found between the TIL counts after NAT and age, postoperative staging, cT, cN, distant metastasis, ER and PR status, or Ki-67 index. We have reached conclusions inconsistent with previous studies regarding the relationship between TILs post-NAT and the clinicopathological characteristics. The different TIL evaluation criteria, including only HER2-positive breast cancer types, and the heterogeneity of the histopathological tissues of HER2-positive breast cancer have likely caused the conflicting results.

According to some studies, cytotoxic agents may release tumor antigens and aid in the recruitment of immune cells to the tumor through mediators such as the pro-inflammatory cytokine interferon-c (37). Moreover, by inducing ADCC through immune cells and immunogenic cell death, trastuzumab can increase the density of CD3^+^ and CD8^+^ TILs and enhance the antitumor immune response (38). This laid the theoretical foundation for our research. Our study showed a significant increase in TILs following NAT in all patients, prompting us to speculate that NAT may activate the local antitumor immune status.

Inconsistent with our research, a previous research has shown that, in HER2-positive breast cancer treated with neoadjuvant chemotherapy plus trastuzumab, high grades of TILs in tissues before NAT were associated with a significant improvement in the pCR rate (39). We observed that cN, higher TILs, and PD-L1+TILs in specimens before neoadjuvant therapy, but no other clinicopathological factors, were independent predictors of pCR in NAT. Previous studies have confirmed that PD-L1+TILs are regulated through adaptive mechanisms and reflect preexisting immunity, and their expressions may be caused by an organism’s strong primary cytotoxic immune attack on tumor neoantigens (34, 40, 41). Therein, chemotherapy and targeted therapy-induced cellular damage and immunogenic cell death will cause a cascade of cellular immune responses, the development of new immunogenic epitopes, antigen cross-presentation, cytokine and chemokine secretion, induction of tumor-specific cytotoxic T cells, and activation of dendritic cells. Similarly, chemotherapy and targeted therapy can also cause a cascade of humoral immune responses (13, 36, 42). This supported a previous theory that chemotherapy and targeted therapy could improve treatment efficacy by increasing the immune activity of patients (43). Furthermore, the FinHER Study showed that the high level of stromal TILs at diagnosis predicted the benefits of trastuzumab adjuvant therapy and proposed that the establishment of a HER2 signal might be the reason for the maintenance of the immunosuppressive microenvironment, However, trastuzumab may break the hypothesis of such an immunosuppressive microenvironment (19). Further research is urgently needed to investigate the relationship between neoadjuvant trastuzumab plus chemotherapy and the immune microenvironment of HER2-positive breast cancer and whether this treatment can affect the immune microenvironment of local antitumor. Besides, our study proved that PD-L1+TILs in pre-NAT specimens were also an independent predictor of pCR in neoadjuvant treatment. One possible explanation for such findings is that the expression of PD-L1 by immune cells, especially TILs, reflects a robust primary immune response and shows an adaptive response to an intensive primary cytotoxic immune attack on cancer neoantigens (44). In conclusion, higher TILs and PD-L1+TILs in pre-NAT specimens may also forecast the effectiveness of neoadjuvant trastuzumab with chemotherapy for HER2-positive breast cancer, in accordance with our findings. However, more research is needed to explicate the antitumor immune response mechanism of TILs and PD-L1+TILs and to clarify the role of PD-L1+TIL in neoadjuvant trastuzumab combined with chemotherapy.

A basic experiment confirmed two main ways to regulate PD-L1 expression after trastuzumab treatment (10). Firstly, the cytokines released by trastuzumab through external pathways may activate trastuzumab-mediated ADCC, thereby upregulating PD-L1 expression on breast tumor cells. Secondly, a trastuzumab-mediated intrinsic pathway to inhibit HER2 downstream cell signal transduction downregulates PD-L1 expression on tumor cells. These pointed out that this extrinsic pathway is related to trastuzumab resistance and that the internal pathway is related to the antitumor immune effect of trastuzumab. The results of the basic experiment may explain the following conclusions we have reached. In our subgroup analysis, PD-L1+TC in the neoadjuvant trastuzumab plus chemotherapy subgroup was significantly reduced, and the results were statistically significant. However, no statistically significant reduction in PD-L1+TC was found in the general population and the subgroup of neoadjuvant chemotherapy alone. This result indicates that neoadjuvant trastuzumab plus chemotherapy may affect the expression of PD-L1+TC through the intrinsic pathway mediated by trastuzumab. Moreover, a study has shown that PD-L1+TC can mediate antitumor immune escape (45). In our study, only the subgroup of trastuzumab combined with chemotherapy showed a decrease in PD-L1+TC after treatment, suggesting that PD-L1-TC is related to the efficacy of neoadjuvant trastuzumab plus chemotherapy. However, the relationship between PD-L1+TC and trastuzumab in truncating tumor immune escape needs further confirmation by basic experiments.

Professor Arlene H. Sharpe has shown that PD-L1 on highly immunogenic tumor cells is enough to promote tumor immune escape and constrain the tumor lysing ability of CD8^+^ T cells (46). Furthermore, chemotherapy can activate the antitumor immune response. This laid the theoretical foundation for our research hypothesis. We found that, in HER2-positive breast cancer, the TIL counts in post-NAT tissues were increased, but PD-L1+TC was decreased, suggesting that neoadjuvant trastuzumab plus chemotherapy may activate the antitumor immune response, thereby inhibiting tumor immune escape.

Our study has several limitations. Firstly, the low sample size hampered conducting statistical analysis on subtype comparisons and adequately powered multivariate analysis. We also did not investigate other immune-oncologic biomarkers such as CTLA-4 and the expressions of other immune checkpoints in tumor and immune cells. Secondly, the PD-L1 status was based on a single antibody. Due to the significant differences in previous studies using different PD-1/PD-L1 antibodies, our results may be limited by the use of a single antibody. Finally, limited by economic factors, this study only included patients receiving single-target chemotherapy, but failed to show a relationship between TILs, PD-L1+TC, and PD-L1+TILs and the efficacy of neoadjuvant dual-target plus chemotherapy in HER2-positive breast cancer patients. To confirm and endorse our results, larger prospective trials with multi-institution cohorts, homogeneous breast cancer tumor subtypes, and several distinct anti-HER2 treatment regimens are required.

High TILs and PD-L1+TILs in samples prior to NAT and lymph node metastasis can predict the pCR for neoadjuvant treatment in HER2-positive breast cancer patients. Both PD-L1+TILs and TILs were changed in pre- and post-NAT samples of HER2-positive breast cancer, suggesting that the immune microenvironment has a crucial role in neoadjuvant treatment. More studies on the mechanism and prospective clinical verification are required.

## Data Availability Statement

The original contributions presented in the study are included in the article/supplementary material. Further inquiries can be directed to the corresponding author.

## Ethics Statement

The studies involving human participants were reviewed and approved by Shandong Cancer Hospital and Institute. The approval number is SDTHEC 201907003 (Shandong, China). The patients/participants provided written informed consent to participate in this study. Written informed consent was obtained from individual(s) for the publication of any potentially identifiable images or data included in this article.

## Author Contributions

HL and MS were responsible for the study design and concept. MS, YC, JC, SY, QT, and XM performed data acquisition. MS analyzed and interpreted the data. JZ and HY contributed to pathological section reading. MS and HL prepared and edited the manuscript. All authors have read and approved the final version of the manuscript.

## Funding

This work was supported by the Natural Science Foundation of China (grant no. 81902713). Financial support was provided by the Natural Science Foundation of Shandong Province (grant nos. ZR2016HM41 and ZR2015HZ004) and the Key Research and Development Program of Shandong Province (grant no. 2018GSF118089).

## Conflict of Interest

The authors declare that the research was conducted in the absence of any commercial or financial relationships that could be construed as a potential conflict of interest.

## Publisher’s Note

All claims expressed in this article are solely those of the authors and do not necessarily represent those of their affiliated organizations, or those of the publisher, the editors and the reviewers. Any product that may be evaluated in this article, or claim that may be made by its manufacturer, is not guaranteed or endorsed by the publisher.
